# Exploring the functional morphology of the *Gorilla* shoulder through musculoskeletal modelling

**DOI:** 10.1111/joa.13412

**Published:** 2021-02-24

**Authors:** Julia van Beesel, John R. Hutchinson, Jean‐Jacques Hublin, Stephanie M. Melillo

**Affiliations:** ^1^ Department of Human Evolution Max‐Planck‐Institute for Evolutionary Anthropology Leipzig Germany; ^2^ Structure & Motion Laboratory The Royal Veterinary College Hatfield UK; ^3^ Collège de France Paris France

**Keywords:** 3D modelling, adduction–abduction, hominoid shoulder, moment arms, musculoskeletal model, scapula functional morphology, western lowland gorilla

## Abstract

Musculoskeletal computer models allow us to quantitatively relate morphological features to biomechanical performance. In non‐human apes, certain morphological features have long been linked to greater arm abduction potential and increased arm‐raising performance, compared to humans. Here, we present the first musculoskeletal model of a western lowland gorilla shoulder to test some of these long‐standing proposals. Estimates of moment arms and moments of the glenohumeral abductors (deltoid, supraspinatus and infraspinatus muscles) over arm abduction were conducted for the gorilla model and a previously published human shoulder model. Contrary to previous assumptions, we found that overall glenohumeral abduction potential is similar between *Gorilla* and *Homo*. However, gorillas differ by maintaining high abduction moment capacity with the arm raised above horizontal. This difference is linked to a disparity in soft tissue properties, indicating that scapular morphological features like a cranially oriented scapular spine and glenoid do not enhance the abductor function of the gorilla glenohumeral muscles. A functional enhancement due to differences in skeletal morphology was only demonstrated in the gorilla supraspinatus muscle. Contrary to earlier ideas linking a more obliquely oriented scapular spine to greater supraspinatus leverage, our results suggest that increased lateral projection of the greater tubercle of the humerus accounts for the greater biomechanical performance in *Gorilla*. This study enhances our understanding of the evolution of gorilla locomotion, as well as providing greater insight into the general interaction between anatomy, function and locomotor biomechanics.

## Graphical abstract

In non‐human apes, certain morphological features have long been linked to greater arm abduction potential and increased arm‐raising performance, compared to humans. Here, we present the first musculoskeletal model of a western lowland gorilla shoulder to test some of these long‐standing proposals. We find that of the glenohumeral abductors only supraspinatus is mechanically enhanced in gorillas, and the deltoid partially changed to an adductor action.

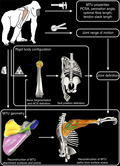

## 1 INTRODUCTION

It is generally accepted that non‐human apes possess adaptations to arboreal environments that are not shared by humans. While arboreal adaptations can be found throughout the ape skeleton, they are thought to be particularly important in the shoulder. In relation to their orthograde body plan, hominoid shoulders sit more dorsally and further apart on the transversely broad thorax compared to quadrupedal monkeys and most quadrupedal mammals (Cartmill & Milton, 1977; Ward, 2007). This shift in position potentially allows for a wider range of upper limb motion, which enables the upright hand‐assisted locomotion that most hominoids display in arboreal contexts. Apes further share a unique set of morphological features in the shoulder that are traditionally understood as reflecting adaptations to their arboreal environments.

**FIGURE 1:**
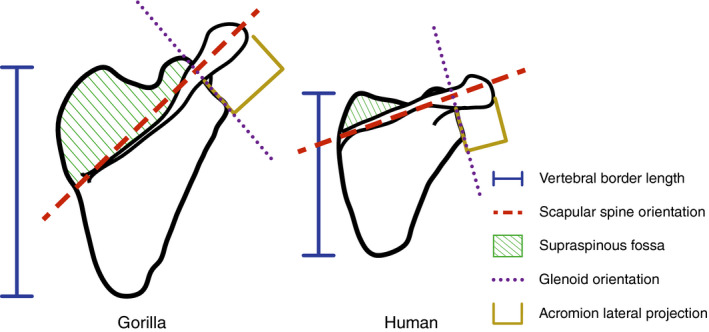
Differences in scapular morphology between gorillas and humans. The superoinferiorly elongated blade (along vertebral border), cranially oriented scapular spine, increased size of the supraspinous fossa, cranial orientation of glenoid and lateral projection of the acromion are understood as signals for suspensory adaptation in gorilla, and their absence in human as a signal for the loss of this adaptation

Correlational studies of functional morphology have shown that primates sharing a given mode of locomotion also share a particular suite of morphological features in the shoulder. Non‐human apes use vertical climbing and forelimb suspension to navigate arboreal habitats and share the following skeletal features: cranially divergent clavicles, a cranially oriented scapular spine, acromion and glenoid, a laterally projecting acromion, a superoinferiorly elongated blade, a medially positioned inferior angle and a relatively large supraspinous fossa (Ashton & Oxnard, 1964; Ciochon & Corruccini, 1977; Corruccini & Ciochon, 1976; Melillo, 2016; Miller, 1932; Oxnard, 1967; Roberts, 1974; Young, 2008). These same features occur in distantly related monkey species that are especially suspensory (Jenkins et al., 1978; Larson, 1995, 1998), but not in humans or other primarily terrestrial primates (Figure 1). This co‐occurrence of form and function provides circumstantial evidence of adaptation to arboreality, especially given the commonality of convergence. Accordingly, studies from the late 19th through mid‐20th centuries commonly characterized all non‐human apes as brachiators.

However, there is a growing recognition that the shoulder form‐function link is not as tight as once believed within apes (Larson, 2015; Larson & Stern, 2013; Melillo, 2016; Taylor, 1997). Gorillas are members of the superfamily Hominoidea and exhibit the shoulder features described above that are traditionally understood as adaptations to below‐branch forelimb suspension. However, forelimb suspension is employed infrequently in adults. Arboreal postural behaviours for adult gorillas mostly consist of resting and feeding. The actual arboreal locomotion is confined to rare events of climbing in and out of food trees using vertical climbing and of traveling between feeding sites within trees by brachiating and orthograde clambering (Crompton et al., 2010). Juveniles between about 5 months and 2 years spend half or more of their time in trees, exhibiting climbing and suspension behaviours. However, they become full quadrupedal knuckle‐walkers after the age of four, when the relative frequency of arboreal locomotion declines to nearly zero (Doran, 1997). Furthermore, the relative amount of time spent in arboreal contexts differs between gorilla subspecies, with mountain gorillas spending substantially more time on the ground than lowland gorillas. Particularly, lowland gorillas display a 20% frequency of the locomotor modes vertical climbing and descent, compared to 1% in mountain gorillas (Crompton, 2016; Crompton et al., 2010). However, this difference in locomotor and positional behaviour is not reflected in bone morphology: these taxa exhibit little to no difference in scapular features linked to arboreal adaptation (Taylor, 1997). Thus, *Gorilla* is no longer recognized as an especially suspensory genus, despite exhibiting the characteristic morphology of the suspensory shoulder and forearm—a circumstance that has been labelled the gorilla paradox (Remis, 1998).

Shoulder morphology reflects a complicated interplay of functional signal and phylogenetic inertia in apes. The confounded functional and phylogenetic division in this group makes it difficult to separate morphological features indicating arboreal adaptation from features that simply reflect membership in the Hominoidea. Enhancing our understanding of the biomechanical benefits of ape shoulder morphology to arboreality is crucial for better disentangling these compound signals.

### 1.1 Previous proposals

Researchers have proposed a number of ideas on how ape shoulder morphology could be biomechanically beneficial in an arboreal context. Despite the knowledge that multiple shoulder functions play a role during suspension (like propulsion and stabilization), ideas discussing how shoulder morphology is advantageous have focused on the specific action of arm‐raising (see Larson, 1993 and references therein). The focus lies on arm‐raising because all arboreal postural behaviours, including vertical climbing and forelimb suspension, require the habitual use of the arm in an over‐head position (Ashton & Oxnard, 1964). Furthermore, in gorillas and other non‐human apes, powerful arm raising may be especially important due to their unique body plan. Their upper limbs are long (especially the forearm and hand) and heavy relative to overall body mass (Zihlman, 1992). Because a greater mass is located in distal segments, a larger inertial load must be overcome to raise the arm. The idea that arm‐raising is an especially important mechanism during brachiation is further supported by the highly influential findings of Ashton and Oxnard (1964). Their results indicated that the main skeletal differences between brachiators and non‐brachiators are related to the functional muscle group responsible for arm elevation. Moreover, Ashton and Oxnard (1963) found that these muscles are relatively more powerful in brachiators than in quadrupedal primates. Thus, apes may be expected to display adaptations that relate to a strong arm‐raising mechanism.

In humans, arm‐raising occurs by the combined movements of scapulothoracic rotation and glenohumeral elevation, a mechanism also referred to as scapulohumeral rhythm (Codman, 1934; Inman et al., 1944; Lucas, 1973). The muscles that function as scapular rotators, most importantly trapezius and serratus anterior, originate from the vertebral column and the ribs, and insert onto the scapular spine and medial border through the inferior angle. The main glenohumeral abductors, the deltoid and supraspinatus muscles, originate from the clavicle, scapular spine, acromion and supraspinous fossa and insert onto the deltoid tuberosity and greater tubercle of the humerus. Because the scapula serves as the primary anchor for muscles involved in arm raising, functional morphologists have focused on explaining how scapula shape affects the function of the glenohumeral abductors and scapular rotators.

However, proposed explanations are vague and sometimes contradictory. Miller (1932) suggested that the cranial orientation of the scapular spine aids the arm‐raising mechanism by providing advantageous attachment locations to the glenohumeral abductors. Roberts (1974) similarly emphasized that the cranially “swept” acromion of non‐human apes improves leverage for the deltoid muscle. Corruccini and Ciochon (1976) suggested that the lateral projection of the acromion, which was reported to be greater in *Gorilla* than in *Homo* (Ciochon & Corruccini, 1977), serves as a beneficial biomechanical arrangement for deltoid and trapezius muscles. In contrast, Ashton and Oxnard (1964) proposed that a cranially oriented scapular spine and elongated scapular blade enhance the function of the scapular rotators, rather than the glenohumeral abductors. This commonly cited explanation implies that the scapular rotation mechanism is expected to be more mechanically advantageous in non‐human apes than it is in humans. However, there are indications that scapula rotation either does not occur during arm‐raising in non‐human apes (Tuttle & Basmajian, 1977) or that the mechanism differs from that documented in humans (Larson et al., 1991). Tuttle and Basmajian (1977) measured muscle activity of the cranial trapezius and caudal serratus anterior, which are thought to be the most important scapular rotators in humans, but found little to no activity in non‐human apes during arm elevation. They concluded that, due to a more cranially oriented glenoid, the scapula already faces cranially, similar to the fully rotated scapula in humans, and therefore apes would have less need for an active scapular rotation mechanism. In contrast, Larson et al. (1991) conducted similar experiments and found that caudal serratus was active during arm‐raising motions. Therefore, Larson et al. proposed that caudal elongation and narrowing of the blade enhance serratus anterior leverage and thereby improve the scapular rotation mechanism (Larson, 2015; Larson et al., 1991), while spine orientation instead reflects an improved shoulder stabilization role of infraspinatus or a structural reinforcement of the scapular blade (Larson & Stern, 2013). However, none of these proposals have been biomechanically tested to date.

Testing these proposals has been challenging for various reasons. Generally, precisely measuring biomechanical parameters that relate to improved leverage or muscle mechanical efficiency is difficult in living animals, and requires invasive methods like marker‐based radiography (An et al., 1984). To avoid these methodological issues, measurements are often taken from cadavers (An et al., 1984; Michilsens et al., 2010). However, as biomechanical properties such as leverage may be dependent on joint angles, the measurements have to be taken for different joint configurations and therefore the methods are often difficult and time‐consuming (Channon et al., 2010; Murray et al., 1995). Furthermore, strong ethical restrictions apply to working with living apes and cadavers are difficult to acquire. Therefore, new methods were favoured to measure biomechanical parameters for locomotion patterns in non‐human apes. Recent advances in the construction and analysis of musculoskeletal computer models provide such new opportunities to study biomechanical structures and relate their function to advantages in biomechanical performance (Seth et al., 2018).

### 1.2 Musculoskeletal Modelling

Musculoskeletal models can be used to calculate the biomechanical properties that relate to enhanced muscle function, like moment and moment arm (Seth et al., 2018). These models are computer‐based, often three‐dimensional, representations of the musculoskeletal system that offer a way to understand performance capability. The models are grounded in mechanics as well as anatomy, thereby building upon the traditional approach of comparative functional morphology. Furthermore, the interactive virtual modelling approach permits the isolation of specific aspects of skeletal geometry or muscle properties to discern their impact on joint mechanics, without the involvement of live animals (Brassey et al., 2017; Hutchinson et al., 2005, 2015).

The total amount of rotational force produced about a joint (moment) is the result of the force produced by a muscle‐tendon unit (MTU) during activation, multiplied by its moment arm. Musculoskeletal models include information about the architectural properties of MTUs and musculoskeletal geometry. Architectural properties like physiological cross‐sectional area, fiber length, pennation angle and tendon slack length determine the force production capability of a MTU (Delp et al., 2007; Delp & Loan, 2000; Hutchinson et al., 2015), whereas musculoskeletal geometry (i.e., muscle origin, insertion and path) affects a MTU’s line of action and thus its distance from the joint rotation centre. The latter parameters determine moment arm, which quantifies how efficiently the linear force produced during muscular contraction is converted to moment. In this way, moment arm provides a measure of colloquial terms like (bio)mechanical advantage and leverage (Sherman et al., 2013). As moment combines effects of properties internal to a MTU with its external geometry, the parameter can be used as a measure of the functional capacity of a musculoskeletal system. Furthermore, the sign of moment arm, and consequently also of moment, indicates whether a muscle would act to increase or decrease joint angles and thus approximately predicts the function (e.g. abductor/adductor) of a muscle (Pandy, 1999), although there are important complexities in this prediction (Kuo, 2001). Because moment arms are strongly influenced by bone shape, size and (musculo)skeletal configuration, this parameter can provide insight into how aspects of skeletal morphology serve to alter forces generated by soft tissues.

In this study, we test the hypothesis that the musculoskeletal configuration of the shoulder in gorillas improves the biomechanical performance of the glenohumeral abductors over arm abduction, compared to humans. We investigate these issues by comparing moment arm and moment production potential of the deltoid, supraspinatus and infraspinatus between musculoskeletal models of the gorilla and human glenohumeral joints. We expect to find higher moment production capabilities in the gorilla model, reflecting an arm‐raising mechanism that is stronger than in humans. We further expect that the osteological features discussed above (cranially divergent clavicles, cranially oriented scapular spine and laterally projecting acromion, etc.) will increase abductor moment arms. Higher moments correlated with higher moment arms would support previous ideas linking differences in skeletal morphology to functional enhancement, and thus the general inference that these features are adaptations to arm‐raising.

## 2 MATERIALS AND METHODS

**FIGURE 2:**
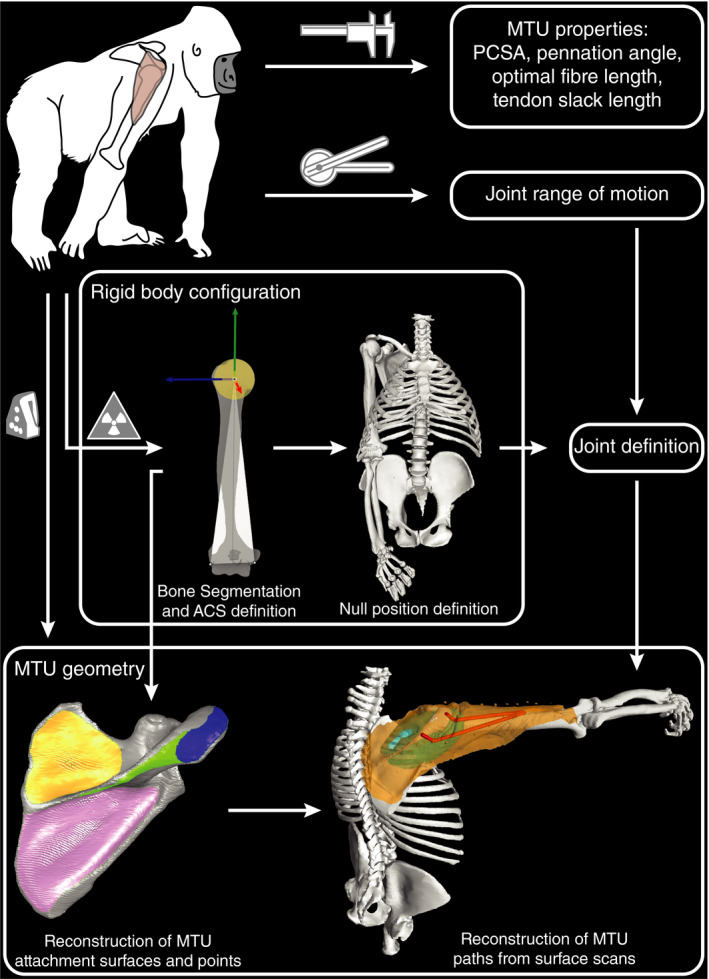
Organisational workflow of data acquisition and model construction. ACS, anatomical coordinate system; MTU, muscle‐tendon unit; PCSA, physiological cross‐sectional area

The gorilla musculoskeletal model was built for use in the open‐source software OpenSim (Delp et al., 2007; Seth et al., 2018). We also used OpenSim to make alterations to an existing human model (Seth et al., ,^2019^, ^2016^) and to analyse the muscle moment arms and moment capacities of both models that are possible during glenohumeral abduction. The gorilla musculoskeletal model was informed by specimen‐specific data (Figure 2), which was collected during a dissection that took place in April 2019. The skeletal input derived from a three‐dimensional reconstruction of a CT‐scan. Rotational degrees of freedom were measured on the cadaver during passive manipulation of the glenohumeral joint, to limit the range of motion of the virtual joint in the model. MTU geometry (attachment sites and paths) was reconstructed by three‐dimensional surface scans taken before and after muscles were removed. The MTU parameters informing the MTU’s force properties were measured, calculated and estimated from data collected during the dissection.

### 2.1 Components of a musculoskeletal model

Musculoskeletal models in OpenSim are based on three primary components: rigid bodies, joints and forces. Rigid bodies contain representations of individual bones or of articulated sets of bones. A three‐dimensional anatomical coordinate system (ACS) is applied to each body, providing a framework to describe the position and orientation of bones, muscles and joints. Moreover, the rigid bodies are hierarchically structured. Bodies situated lower in the hierarchy (child bodies) sit within the coordinate system of bodies situated higher in the hierarchy (parent bodies). Joints define the possible movement of a child body relative to its parent. Each joint is composed of a joint‐specific coordinate system (JCS), of coordinates that restrict the joint excursion (up to three rotational and three translational degrees of freedom) and of functions that define the movement path. Forces are able to control the movement of rigid bodies around the connecting joints and are informed by the geometry and properties of each MTU. The geometry is composed of a MTU’s origin and insertion points and of a MTU’s path, which is constrained by path points and wrapping surfaces. The parameters fibre length, pennation angle, physiological cross‐sectional area and tendon slack length are used to scale the generic Hill‐type muscles (Delp & Loan, 2000; Zajac, 1989). Because the goal of this study is to compare a human and a gorilla model, care was taken to build our gorilla model in a manner that maximizes comparability with the existing human model (Seth et al., 2019, 2016).

### 2.2 Data acquisition

All observations informing the model came from the same female western lowland gorilla (*Gorilla gorilla*). The gorilla was a zoo animal, euthanized at 48.8 years (December 2012) after suffering from age‐related ailments (heart failure and kidney problems). The dead mass was 80.5 kg. The fresh‐frozen cadaver (stored at −20°C) was acquired ethically through collaboration with the Cleveland Museum of Natural History, Erie Zoo and Cleveland Metroparks Zoo. Data collection procedures and muscle property calculations are based on Hutchinson et al. (2015), with minor modification.

### 2.3 Rigid body configuration

**FIGURE 3:**
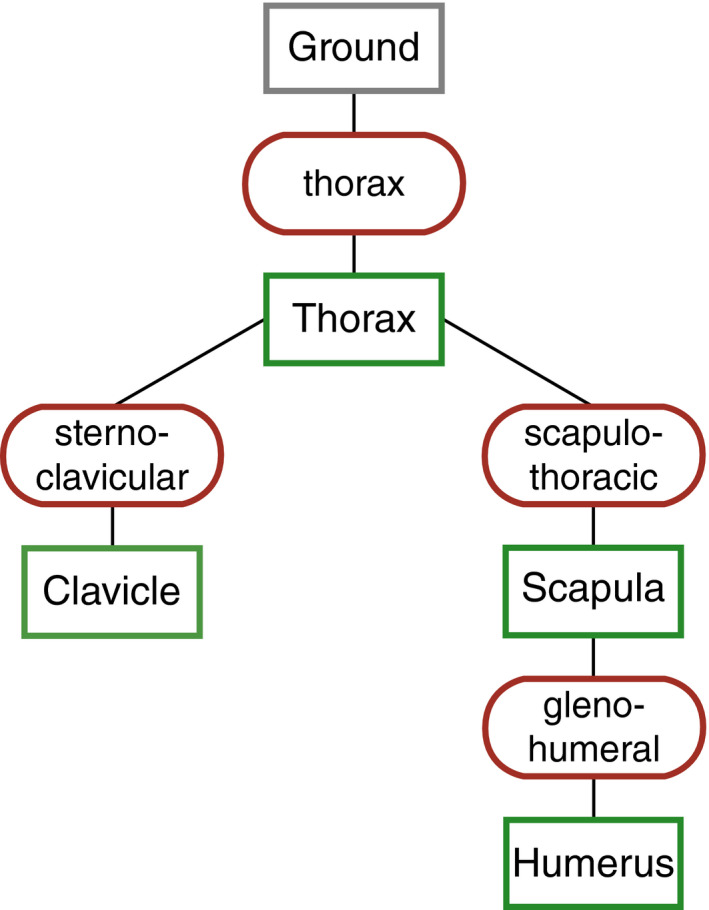
Composition of our gorilla musculoskeletal shoulder model. In the hierarchical chain of the model, joint names are lowercased in red circles, rigid body names are capitalized in green boxes. The hierarchy follows from top to bottom, with each child rigid body sitting below its parent rigid body. The rigid bodies Thorax, Clavicle and Scapula are held immobile in our analysis

The specimen was CT scanned at the Ohio State University College of Veterinary Medicine using a Revolution Evo Lightspeed CT (GE Healthcare), prior to dissection. The scan (voltage: 120 kV; current: 319 mA) contained 1843 slices (voxel size: 0.977 × 0.977 × 0.625 mm). Bones of the upper limb and thorax were manually segmented in Avizo software (version 9.3.0, Visualization Sciences Group), using a thresholding approach. Meshes were decimated and smoothed in Geomagic Studio® (version 2013, RSI 3D‐Systems). The model is composed of four rigid bodies: thorax (represented as a single body), clavicle, scapula and humerus (Figure 3). Meshes of the lower arm (radius, ulna and hand) are included for visualization purposes only.

ACSs were defined for thorax, clavicle, scapula and humerus in Rhinoceros software (version 6, McNeel Europe). Coordinate centre and orientation of axes are based on specific landmarks as described in the ISB recommendations (Wu et al., 2005; also see Seth et al., 2019). Following the recommendations, a sphere was used to define the centre of the humerus ACS (Figure 2) in a geometrically defined and semi‐automatic manner via Matlab code (Bishop et al., 2020). The meshes with defined ACSs were next transferred into Maya software (version 2019, Autodesk), where the meshes were arranged hierarchically and the null position was defined (with humerus long axis in parallel to sagittal plane). These inputs were written into an OpenSim musculoskeletal model file using a Matlab script (Bishop et al., 2020). Rigid body hierarchy, ACS definitions and null positions are identical between gorilla and human models.

### 2.4 Joint definition

The shoulder complex consists of four different joints that describe the movement of the rigid bodies against each other (Figure 3). Their setup follows the ISB recommendations (Wu et al., 2005). In this study, analyses were carried out with all rigid bodies except the humerus kept immobile. In this way, we investigate performance of the glenohumeral joint while controlling for potential interspecific differences in scapulothoracic rotation during arm elevation. As recommended by the ISB, the centre of the glenohumeral JCS is identical to the centre of the humerus ACS. Movement about the joint has three rotational degrees of freedom. The way that movement is described follows the ISB recommendations (Wu et al., 2005), which differ slightly from traditional anatomical terminology. Together, glenohumeral elevation and plane of elevation specify the amount of arm elevation and in which plane the elevation occurs. As our focus lies on abduction and adduction, all analyses of glenohumeral elevation were conducted in a plane of elevation of 0°, which is roughly equivalent to the coronal plane. Axial rotation specifies internal/external (long axis) rotation and was kept at 0° as well.

Joint excursion measurements were collected after the cadaver was thawed (24 h at temperatures between 5° and 15°) and before dissection began. The scapula was manually stabilized on the thorax by one member of the dissection team while the orientation of the humerus was manipulated. A goniometer was placed at the glenohumeral joint centre to measure passive range of motion (Hammond, 2014; Norkin & White, 2016). The limit of mobility was determined when a certain (admittedly subjective) degree of resistance was met, with care taken to avoid joint damage. Measurements were repeated three times and the mean was taken. The glenohumeral joint in the model was configured to resemble both the joint range of motion recorded during the dissection and the human model (Seth et al., 2019).

### 2.5 Muscle‐tendon units

MTUs represent either a whole muscle or functionally distinct portions of a muscle, which can be useful when muscles have broad attachment sites. The MTUs characterized in our model and the corresponding MTUs of the human model are listed in Table 1. Decisions about how to model muscles were based on anatomical and experimental observations that suggest different functional roles for different fibre groups (Diogo et al., 2011; Gilroy et al., 2012; Larson & Stern, 1986; Larson et al., 1991; Netter, 2010). To maintain consistency, the abbreviations for the deltoid subunits used in the gorilla model are used to refer to the corresponding subunits of the human model as well.

**TABLE 1:** Functional divisions of muscle gross anatomy are reported with corresponding muscle‐tendon unit (MTU) abbreviations used in the gorilla shoulder model. Abbreviations for human model MTU’s follow the names given by Seth et al. (2019)

Muscle (gross anatomy)	MTUs of Gorilla model	MT subunits of gorilla model	Number MTUs	MTUs of Human model	MT subunits of Human model	Number MTUs
Deltoid	Delt		3	Deltoideus		3
Clavicular part		clav	1		Clavicle_A	1
Acromial part		acro	1		Scapula_M	1
Spinal part		spin	1		Scapula_P	1
Supraspinatus	Supraspin		1	Supraspinatus		2
Superior		–	–		A	1
Inferior		–	–		P	1
Infraspinatus	Infraspin		1	Infraspinatus		2
Superior		–	–		S	1
Inferior		–	–		I	1

The supra‐ and infraspinatus muscles are divided into two subunits in the human model, with one subunit having an origin positioned more superiorly and the other more inferiorly within the supra‐ and infraspinous fossae, respectively. We represent these muscles as single units in the gorilla model, with the origin positioned in the centre of their respective attachment site. Because moment arm is determined by the spatial configuration of the muscle attachments and paths relative to the joint centre, the intermediate origin location of the gorilla muscle units would be expected to result in a moment arm intermediate between the two human subunits (with other factors held constant). In the Results section, we depict the human supra‐ and infraspinatus moment arms as averages of the two subunits for ease of interspecific comparison. The results with separated subunits are reported in the Supporting Information. Differences in muscle division also affect moment curves, particularly because the mass of a complete muscle is separated into smaller units. To address this issue, the human supra‐ and infraspinatus moment curves are presented as the sum of both subunits of each muscle. In addition, we used sensitivity analysis to investigate the effects of these differences in supraspinatus and infraspinatus attachments (Text S1, Figures S2 and S3).

### 2.6 MTU geometry

Observations on MTU attachments and paths were recorded in surface scans conducted during the dissection (for more information see Text S2). First, muscles were identified with reference to Netter (2010); Diogo et al. (2011); and Gilroy et al. (2012). Next, coloured pins were used to label each muscle unit's origin, insertion and midline. Additional pins were used to label palpable osteological landmarks on each rigid body. After pinning, we used a structured‐light surface scanner (Artec Space Spider with Artec Studio 12 software, Artec 3D) to collect three different digital representations of the dissection surface: (a) with the glenohumeral joint fully abducted, (b) fully adducted and (c) in an intermediate position (not quantified). In this way, the three scans capture the change in muscle paths throughout shoulder excursion, as well as an impression of kinematic sequences throughout passive manipulation (Figure S5). The surface scanning process was repeated as the dissection proceeded through progressively deeper muscle layers. Surface scans collected during the dissection were registered to the CT scan and model using the osteological landmarks (Figure S4). A centroid was calculated for each attachment surface and this (*x*, *y*, *z*) point served as the MTU’s origin or insertion. As a result, the specimen‐specific muscle attachment sites and pathways observed during dissection could be transferred directly into the model building space (details on the procedure are given in the Supporting Information).

If unconstrained, muscle units running directly from origin to insertion may bisect bones or take paths that are unrealistic for other reasons, because bones are only visualization objects rather than obstacles in the software environment. In OpenSim, this issue is addressed with the insertion of path points (fixed, via or moving) and wrapping surfaces, which constrain the paths muscles take in the null position and throughout joint excursion. We employed two approaches to constraining muscle paths. First, we inserted the same wrapping surfaces and path points present in the existing human model (Seth et al., 2019) into our gorilla model, and then reshaped these features to reflect the morphology of the gorilla skeleton. Next, we examined the resulting muscle paths and compared them to the surface scans for the three different arm elevation positions. To achieve the latter comparison, the dissection surface scans were registered to the model building space. The modelled joint was then moved to imitate the cadaveric glenohumeral positions (Figure S5). This procedure ensured that muscle paths specified in our model corresponded to dissection observations, across joint excursion, for the same specimen. A list of wrapping surfaces used is given in Table 2.

**TABLE 2:** Muscle wrapping surfaces used in the model, with dimensions

Muscle(s)	Rigid body location	Shape	Dimensions (m)		
			A	B	C
Delt (all)	Scapula	Ellipsoid	0.0465*	0.1007*	0.0496*
Infraspin	Scapula	Ellipsoid*	0.0465	0.1007	0.0496
Infraspin	Humerus	Ellipsoid	0.0306*	0.029*	0.0277*
			Radius	Length	**–**
Supraspin*	Scapula	Cylinder	0.015	0.0555	–
Supraspin	Humerus	Sphere	0.0289*	–	–

Muscles that use the wrapping surfaces to restrict their paths are given in the first column, (all) refers to all MTUs of a specified muscle.

The asterisk (*) indicates differences to the wrapping surface configuration used in Seth et al. (2019). In their model, the supraspinatus pathway is not constrained by a further wrapping surface attached to the scapula and the infraspinatus wrapping surface on the scapula is a cylinder. Some dimensions were further adjusted to mirror the gorilla specific anatomy.

To minimize the influence of allometric scaling, all moment arm lengths (m) and moment values (Nm) are normalized by humerus length (m). The humerus length used in the models was determined as 0.359 m for the gorilla and 0.326 m for the human. Non‐normalized moment arms are reported in the Supporting Information. Ideally, moment values would additionally be normalized by body mass. Unfortunately, body mass was not available for the human specimen (Klein Breteler et al., 1999).

### 2.7 MTU properties

The four parameters determining the MTU properties are optimal fibre length ($l_{0}^{m}$), pennation angle (θ), muscle physiological cross‐sectional area (*A*_phys_) and tendon slack length ($l_{s}^{t}$). The first three came from dissection, as follows. Tendon slack length ($l_{s}^{t}$).was estimated following the equations of Manal and Buchanan (2004) and using the approach reported in Heers et al. (2018). The estimations are based on MTU length ranges of motion of all degrees of freedom of each joint a muscle spans. OpenSim's Muscle Analysis plotting tool was used to estimate the muscle‐tendon length ranges for each MTU of our musculoskeletal model. Muscle pennation angle (θ) influences muscle cross‐sectional area and muscle force calculation only for highly pennated muscles (>20°, Carlson, 2006; Thorpe et al. 1999; Zajac, 1992). During dissection, deltoid, supraspinatus and infraspinatus muscles were found to not be highly pennate. Therefore, a value of 0° was used as input into the musculoskeletal model. Pennation angle is accounted for in the muscle geometric calculations intrinsic to OpenSim's implementation of a Hill‐type muscle model and is therefore not included in *A*_phys_ calculations (Bishop et al., 2020; Zajac, 1989). After photographs (with visible scale) were taken to measure muscle‐tendon length (±0.1mm), the tendon was removed and muscle belly mass of each muscle was measured using an electronic balance (±0.01 g). Subsequently, the muscle bellies were digested in 20% nitric acid solution for 24–48 h, a process that also visibly reduced the muscle length (shrinkage). Intact muscle fibres were gently separated and transferred into glycerine‐coated petri dishes to terminate the digestion process. The lengths of 10–20 muscle fascicles were measured for each muscle unit on digital photographs, using the measure function in Fiji software (Schindelin et al., 2012). The average of these measured muscle fascicle lengths was corrected for a shrinkage of 43% (Alway et al., 1989; Heron & Richmond, 1993) that was introduced by the nitric acid digestion. This approach resulted in fascicle lengths comparable to those reported in similar studies using different approaches (Table S2). The corrected muscle fascicle length value (L) was assumed to be equivalent to optimal fibre length ($l_{0}^{m}$; Zajac, 1989). Averaged muscle masses (*m*_musc_) and fascicle lengths (L) were calculated for each MTU. We used a muscle density (d) value of 1.06 × 10^3 ^kg/m^3^, which has been used as a generalized value for mammalian muscle (Brown et al., 2003; Mendez, 1960; Ward & Lieber, 2005). Muscle cross‐sectional area (*A*_phys_) was calculated using the following equation:

$$A_{\text{phys}}=m_{\text{musc}}{\left(\text{Ld}\right)}^{‐1}$$

We further calculated muscle maximum isometric force capacity *F*_max_ as:

$$F_{\max}=3.0\times 10^{5}m^{‐2}A_{\text{phys}}$$

The constant in this equation is specific muscle tension. Similar values were used in other studies on musculoskeletal models in vertebrates, including hominoids (Goh et al., 2017; Hutchinson et al., 2015; O'Neill et al., 2013; Umberger et al., 2003). However, different equations were used to calculate the MTU properties in the human shoulder model that we use for comparison (Nikooyan et al., 2011). To maintain consistency between models, the equations given above were used to recalculate values for the human model, based on the muscle parameters reported by Klein Breteler et al. (1999) and the MTUs described in Seth et al. (2019) (Table S1).

## 3 RESULTS

### 3.1 Musculoskeletal model parameters

**FIGURE 4:**
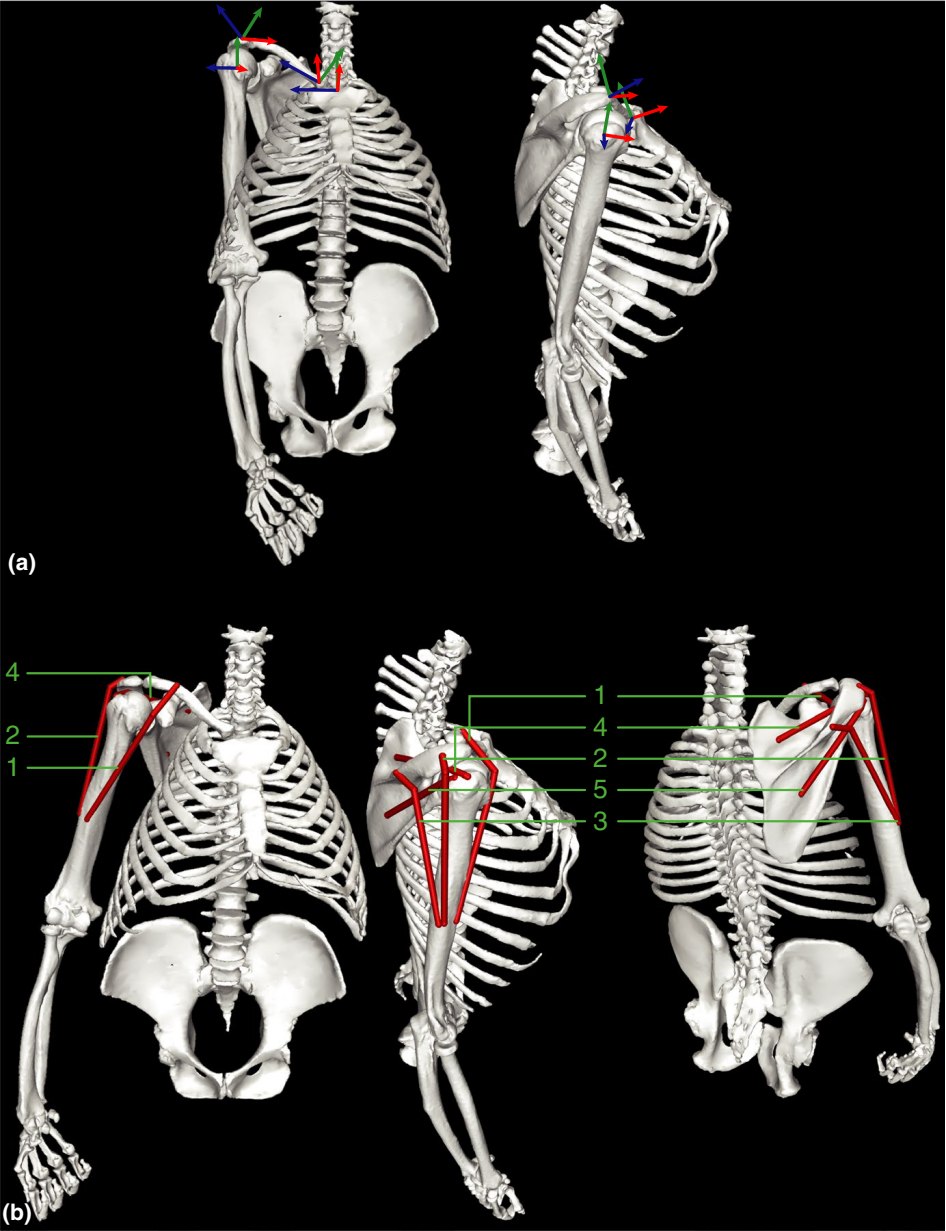
Musculoskeletal model of the gorilla. (a) Zero (reference) position of the model, view from frontal (left) and lateral (right). The three axes of the rigid body coordinate systems (ACSs) of the humerus, scapula, clavicle and thorax are displayed, with *X*‐axis in red, *Y*‐axis in green and *Z*‐axis in blue. The origin of these coordinate systems coincide with the origin of the corresponding JCSs. Clavicle ACS not visible in lateral view, for clarity. (b) Muscle‐bone configuration of the model, in resting position (arm elevation of 15°). View from frontal (left), lateral (middle) and back (right). Muscles are represented as red bands. 1. Clavicular Deltoid, 2. Acromial Deltoid, 3. Spinal Deltoid, 4. Supraspinatus, 5. Infraspinatus

The measured and calculated MTU properties are reported in Table 3. The model together with the rigid body coordinate systems is shown in its zero (reference) position in Figure 4a with all joint angles set to zero. Figure 4b shows the model with the arm in a “resting” position (glenohumeral elevation of 15°) with all muscles visible. Positions between zero and resting position (0°–15° glenohumeral elevation) are anatomically not feasible, as observed during the range of motion measurements. Therefore, all following analyses start from the resting position. The arm elevation sequence analysed using the musculoskeletal model is shown in the Supplementary Information (Video S1).

**TABLE 3:** Muscle‐tendon units (MTUs) used in the musculoskeletal model (names abbreviated) with properties used for the final analyses

MTU	Muscle mass, *m*_musc_ (kg)	Fascicle length, L (m)	Pennation angle; *θ* (°)	Physiological cross‐sectional area, *A*_phys_ (m^2^)	Maximum isometric force, *F*_max_(N)	Tendon slack length; $l_{s}^{t}$ (m)
Delt_clav	0.0633	0.1757	0	0.0003	101.92	0.0113
Delt_acro	0.1662	0.0886	0	0.0018	531.12	0.0703
Delt_spin	0.0567	0.1361	0	0.0004	118.00	0.0443
Supraspin	0.0840	0.0662	0	0.0012	358.66	0.0380
Infraspin	0.1048	0.0839	0	0.0012	353.75	0.0294

### 3.2 Moment arm comparison

**FIGURE 5:**
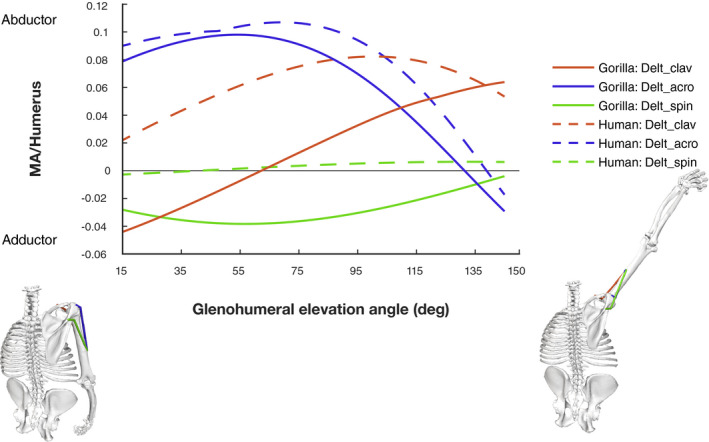
Normalized moment arms of deltoid muscle plotted against glenohumeral elevation. Moment arms are normalized by humerus length. The grey solid line separates MTUs acting as abductors (positive moment arms) from those acting as adductors (negative moment arms). See text for details

The deltoid muscle is the main arm abductor in hominoids, traditionally divided into three different functional units. The normalized moment arms for these three units over arm elevation from the present study and the human model of Seth et al. (2019) are shown in Figure 5 (for absolute values refer to Figure S1). The acromial portion of the deltoid (Delt_acro) shows the greatest similarity in moment arm between species. We found a slightly shorter moment arm in the gorilla model, but with a similarly‐shaped curve over glenohumeral elevation.

The moment arm values of the clavicular deltoid (Delt_clav) are smaller in the gorilla model, and the peak shifted towards higher elevation angles. The gorilla model further predicts that the clavicular fibres of the deltoid change function over arm elevation, with the traditional abductor function achieved for glenohumeral elevation beyond about 60°. In less elevated positions, the model predicts a moment arm that would adduct the arm. In contrast, the human model predicts a retention of abduction function for the clavicular fibres throughout glenohumeral excursion, with the peak occurring between about 95–115° of elevation.

Deltoid fibres attaching to the scapular spine (Delt_spin) maintain moment arm values near zero in the human model. In contrast, the gorilla moment arm values are substantial, non‐linear and predict a pure adductor function for the spinal deltoid.

**FIGURE 6:**
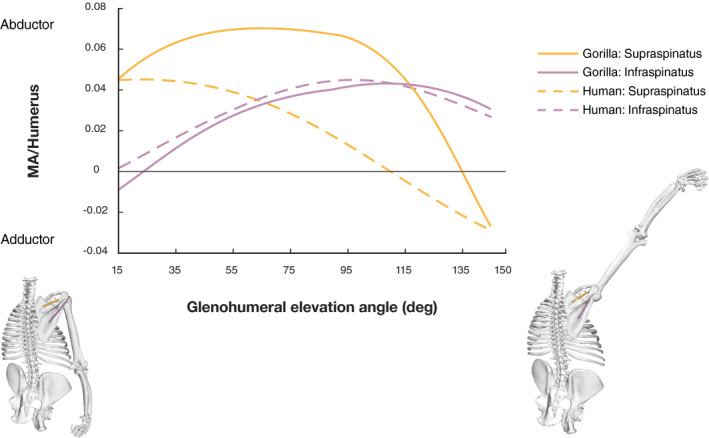
Normalized moment arms of supraspinatus and infraspinatus muscle plotted against glenohumeral elevation. Moment arms are normalized by humerus length. The human model moment arm results each represent the mean of the two subunits. The grey solid line separates MTUs acting as abductors (positive moment arms) from those acting as adductors (negative moment arms). See text for details

The supraspinatus and infraspinatus muscles are components of the rotator cuff. Both muscles enhance arm abduction, external rotation and glenohumeral joint stability. The gorilla infraspinatus moment arm curve is nearly identical to a calculated average of the two human subunit curves (Figure 6) and falls between the curves when the two subunits are plotted separately (Figure S3). Furthermore, sensitivity analyses demonstrate that when the single gorilla infraspinatus origin is moved more superiorly or more inferiorly (to mirror the origins defined for each subunit in the human model), the gorilla curve shifts toward the corresponding human curve (Figure S3). Both models predict an abductor role for this muscle, except at very low elevation angles.

In contrast, the models show a marked difference in supraspinatus moment arm (Figure 6). Abductor moment arm is considerably larger in the gorilla model and the greater magnitude is maintained over a wider range of glenohumeral elevation, only becoming smaller for elevation angles above 95°. In the human model, moment arms are largest at low angles of glenohumeral elevation and reduce consistently. The intermediate moment arm of both subunits loses its ability to act as abductor by 105° of glenohumeral elevation, whereas abductor potential in gorilla is lost only after 135°. Sensitivity analysis shows that the gorilla supraspinatus moment arm remains greater than the human moment arm, despite shifts in origin location (Figure S2).

**FIGURE 7:**
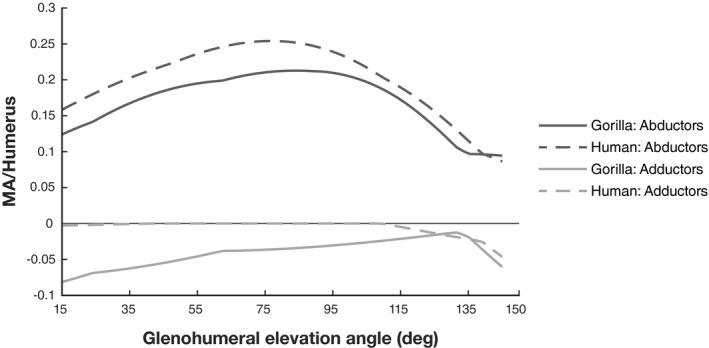
Total abductor and adductor moment arms (MA) normalized by humerus length plotted against glenohumeral elevation. Abductor moment arm is the sum of all positive moment arm values calculated for deltoid, supraspinatus and infraspinatus muscles. Adductor moment arm was calculated using negative moment arm values

The comparison of the total glenohumeral moment arm between the gorilla and human model is shown in Figure 7. While the trend of abductor moment arm changes (the sum of all positive moment arms) is similar in both models, values of the gorilla are generally smaller. Gorilla adductor moment arms (the sum of all negative moment arms) however are generally greater compared to the human model, where values mostly remain close to zero.

### 3.3 Comparison of moment‐generating capacity

**FIGURE 8:**
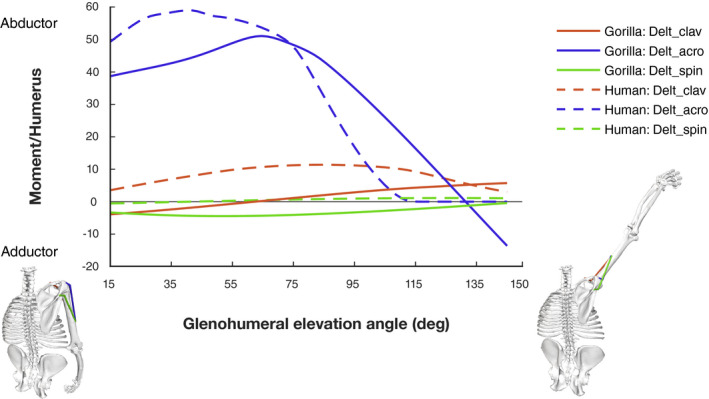
Deltoid moment normalized by humerus length plotted against glenohumeral elevation. The grey solid line separates MTUs acting as abductors (positive moment) from those acting as adductors (negative moment). The gorilla model at the bottom visualizes the arm position at 15° and 145° of glenohumeral elevation, respectively

The acromial deltoid possesses the greatest potential for producing abduction moment in both species (Figure 8). This is primarily due to the architectural properties of these MTUs. In both species, the acromial fibres have much higher maximum isometric force values than the clavicular or spinal portions of the deltoid (see Table 3 and Table S1).

The gorilla and human acromial deltoid moment curves differ in the height and location of their peaks. The results suggest that the human Delt_acro is capable of overall higher abduction moment production, and that maximum moment is achieved early in the elevation sequence. However, the human curve falls off precipitously after about 75° of elevation and is no longer capable of moment production beyond about 115°. The gorilla Delt_acro peak is slightly lower overall, and occurs at a higher joint angle. This MTU retains the ability to generate abduction moment across higher elevation angles, until about 135°.

Interspecific differences in moment curves for the other deltoid units are more subtle and follow a similar trend as the moment arm curves. The clavicular deltoid has a lower moment production potential in the gorilla model and its action potential changes across glenohumeral elevation. Moment production potential of spinal deltoid is low for both species (Figure 8). While moment remains close to zero in the human, moment production potential is slightly higher in the gorilla, where spinal deltoid serves as a pure adductor.

**FIGURE 9:**
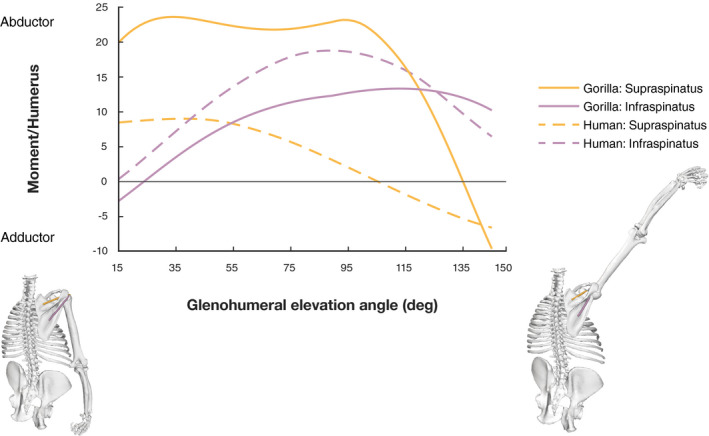
Supraspinatus and infraspinatus muscle moment normalized by humerus length plotted against glenohumeral elevation. The human model moment results each represent the sum of the two subunits’ moments. The grey solid line separates muscle units acting as abductors (positive moment) from those acting as adductors (negative moment). The gorilla model at the bottom visualizes the arm position at 15° and 145° of glenohumeral elevation, respectively

Figure 9 shows moment results for the supra‐ and infraspinatus. The results suggest that the gorilla supraspinatus is capable of generating much greater abduction moments than the human supraspinatus. In the early phases of glenohumeral elevation, the gorilla moments are roughly twice as high as the human values. The human abduction moment production potential decreases thereafter, while the gorilla potential remains high as glenohumeral elevation increases. Between about 70° and 110° elevation, the gorilla supraspinatus moment is up to four times larger than the human values. The marked difference between species stems from the higher maximum isometric force values for the gorilla supraspinatus (see Table 3 and Table S1), amplified by the larger abductor moment arm (Figure 6). Similar to the gorilla supraspinatus moment arm values, moment values remain close to the maximum until a glenohumeral elevation angle of 100°.

With regard to the infraspinatus, the human model suggests higher abduction moment production potential than for *Gorilla*. Within each species, moment production potential of supraspinatus is greater than that of infraspinatus for low elevation angles and vice versa for high elevation angles. However, this transition occurs at 40° of arm elevation in the human model but at 120° in the gorilla model. Furthermore, the species show inverse patterns for supra‐ and infraspinatus abduction moment production. While supraspinatus is a stronger abductor than infraspinatus in *Gorilla*, the contrary is observed in the human model.

**FIGURE 10:**
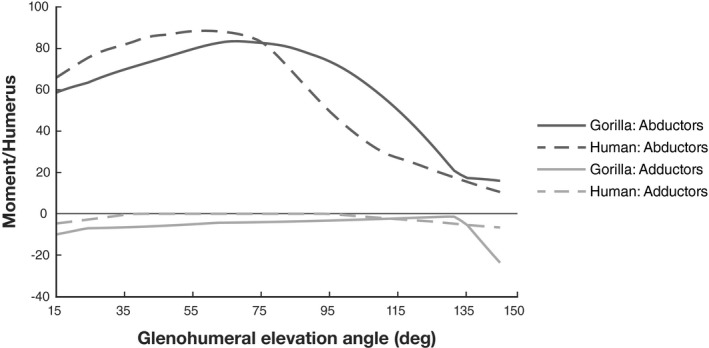
Total abductor and adductor moment potential normalized by humerus length plotted against glenohumeral elevation. Abductor moment potential is the sum of all positive moment values calculated for deltoid, supraspinatus and infraspinatus muscles. Adductor moment potential was calculated from all negative values

The comparison of the total abductor potential (the sum of all positive moments) for deltoid, supra‐ and infraspinatus demonstrates similar maxima between the two models (Figure 10). The primary difference between species lies in the degree of glenohumeral elevation where abduction moments are highest. The abductor potential in the gorilla is less than in the human model for low amounts of arm elevation. Beyond elevation angles of 80°, however, moment production potential is distinctively higher in the gorilla. After elevation angles of 135°, abduction moment potential is small in both models. Total adductor potential (sum of all negative moments) is low for both models throughout their range of elevation motion, but with a slightly higher potential observed in the gorilla.

## 4 DISCUSSION

Skeletal differences between gorillas and humans have been linked to a stronger arm‐raising mechanism in the former species. This study constitutes the first direct test of this idea. Here, we presented the development of a three‐dimensional musculoskeletal model of a gorilla glenohumeral joint, which was built using data on musculoskeletal geometry and MTU properties collected during a dissection. We combined dissection observations with CT and surface scanning to reflect specimen‐specific musculoskeletal properties. The model allows the prediction of MTU moment arm, force and moment production potential for major shoulder muscles crossing the glenohumeral joint. Results from this subject‐specific gorilla model were compared to a human shoulder model, to enhance our understanding of arm‐raising abilities in non‐human apes.

### 4.1 Biomechanical performance of glenohumeral abductors

One objective of our study was to investigate whether the biomechanical capacity of glenohumeral abductors is improved in gorillas, compared to humans. Contrary to expectations, our results suggest that gorillas and humans are capable of producing similarly strong glenohumeral abduction, after differences in humerus length are taken into account (see Figure 10). The primary difference between species relates instead to the joint angles where high moments can be generated. While the arm‐raising performance is similarly strong in gorilla and human, the gorilla is able to maintain higher abductor moments above 90° of arm elevation, compared to the human without scapular rotation.

We further expected to find support for ideas that link differences in scapular morphology to functional enhancement of arm‐raising in gorillas. These hypotheses are cited often in discussions of hominoid shoulder functional morphology (Green, 2013; Larson, 1993; Potau et al., 2009; Selby & Lovejoy, 2017; Shea, 1986; Sonnabend & Young, 2009; Taylor, 1997) and interpretations of functional capabilities in extinct hominins (Harmon, 2013; Larson, 2013; Melillo, 2016; Ward, 2002), but have never been tested. Interspecific differences in bone shape and skeletal configuration are expected to have the most direct effect on moment arm (Smith & Savage, 1956). Therefore, a functional enhancement due to morphological changes would be evident by greater moment arms and linked to greater moment capacities. However, our results suggest that differences in moment production capacity exist between models, despite minimal differences in moment arms. While total abductor moment potential is greater in gorilla for higher elevation angles, overall abductor moment arms are slightly smaller than in the human model (Figure 7). This implies that soft tissue properties (especially *F*_max_) have a great impact on overall glenohumeral abductor capacity. Therefore, our results highlight the importance of including soft tissue properties in biomechanical analyses. Unfortunately, such data collection is often impossible, especially when studying fossils. Thus, caution has to applied in cases where the interpretation of biomechanical capabilities is only based on moment arm results, especially in estimating peak moments (e.g. see discussion in Hutchinson et al. 2005 and Brassey et al. 2017).

### 4.2 Biomechanical performance of deltoid

Non‐human ape morphology was previously understood to enhance function of the deltoid muscle (the main arm abductor) in particular. Specifically, the cranial orientation of scapular spine and greater lateral acromion projection were hypothesized to provide enhanced deltoid leverage (Corruccini & Ciochon, 1976; Larson, 1993; Miller, 1932; Roberts, 1974).

**FIGURE 11:**
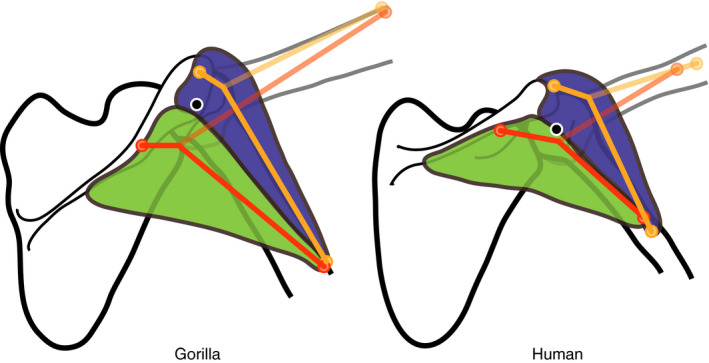
Acromial and spinal deltoid MTU path in gorilla (left side) and human (right side). The distance between glenohumeral rotation centre (black‐white circle) and spinal deltoid MTU path (red line) is greater in the gorilla than in the human model, but similar for acromial deltoid (orange line). Acromial (blue) and spinal deltoid (green) muscles are visible for glenohumeral elevation angles of 35°. MTU paths and humerus shape are displayed for glenohumeral elevation angles of 35° (darker) and 105° (lighter)

However, the spinal deltoid, which originates from the scapular spine and inserts onto the deltoid tuberosity in both species, deviates from the expected abductor action in *Gorilla* (Figure 5). Instead, the negative moment arm values suggest a pure adductor action (a morphological tendency to adduct the arm). This difference in moment arm compared to the human model results from a different line of action. In gorillas, the MTU’s path runs further caudal to the glenohumeral joint centre due to the oblique orientation of the scapular spine and glenoid (Figure 11). Thus, interspecific differences in scapular spine orientation do not appear to enhance the deltoid abductor ability, but instead change the action of the spinal deltoid from a potential abductor to an adductor role, contradicting earlier hypotheses (Miller, 1932).

Our findings are supported by previous observations on muscle activation during various kinds of movement in apes. Larson and Stern (1986) presented electromyography (EMG) measurements of deltoid activation in chimpanzees during vertical climbing and arm‐swinging. The authors found that the activity of the spinal deltoid was out of phase with the other two deltoid units. The clavicular and acromial deltoid were active during phases of arm protraction and abduction during voluntary reaching, vertical climbing, and arm swinging. In contrast, the spinal deltoid was highly active during the support phase and beginning of swing phase, when the arm is already overhead and muscle contraction serves a propulsive function (described by Larson & Stern, 1986 as “propulsive retraction” and adduction). We investigated glenohumeral excursion in the coronal plane, which is only one component of arm movement during arboreal locomotion (Isler, 2005). Future work investigating whether similar differences in moment arm occur during arm elevation in the sagittal plane (pro‐ and retraction) is necessary. Yet, it is encouraging that our modelling results are consistent with EMG studies suggesting a similar divergence in action between the spinal deltoid and the two other deltoid MTUs.

A more laterally projecting acromion was traditionally understood to enhance leverage of the acromial deltoid. Acromial projection has been linked to enhanced arm raising in hominoids compared to monkeys (Corruccini & Ciochon, 1976; Roberts, 1974) and Ciochon and Corruccini (1977) showed that acromial projection is greater in African apes than in humans. However, our results suggest that despite anatomical differences in acromion shape being present between gorillas and humans, moment arms of acromial deltoid are similar in both species (Figure 5). The model comparison indicates that greater acromial projection in *Gorilla* does not increase the distance between glenohumeral joint rotation centre and this MTU’s line of action.

The measurement of acromial projection used by Roberts (1974) and Corruccini and Ciochon (1976), which is called the coraco‐acromial index, quantifies the projection of the acromion lateral to the glenoid, or lateral to a line connecting the tips of the acromion and coracoid (respectively). However, these measurements may fail to capture the structural relationships most relevant to acromial deltoid leverage.

Studies discussing the impact of shoulder morphology on deltoid leverage in humans have long focused on different sets of structural relationships (Howell et al., 1986; Iannotti et al., 1992). These studies have shown that the deltoid moment arm is affected by the amount that the muscle wraps around the humeral head. This wrapping amount is increased where the radius of the humeral head is greater, which is determined laterally by the greater tubercle projection. Therefore, it is not the acromion projection relative to glenoid (Ciochon & Corruccini, 1977; Corruccini & Ciochon, 1976; Craik et al., 2014), but rather the relationship between acromion projection and greater tubercle projection that is more relevant to deltoid leverage (Rietveld et al., 1988). The latter is described by Nyffeler et al. (2006) as the acromion index. This biomechanically important parameter has not been quantitatively addressed in comparative studies across hominoids. Additionally, the radius of the non‐spherical humeral head differs across its circumference due to the protruding greater and lesser tubercles. In the musculoskeletal models, this was addressed by using an ellipsoid wrapping surface for the deltoid (Table 2). As the deltoid spans most of the humeral head in a broad sheet, we expect some variation of wrapping and moment arm across the muscle depending on the path around the ellipsoid. This could be addressed in future analyses by dividing the deltoid MTU into smaller subunits. Future analyses would further benefit from incorporating measurements of scapular and corresponding humeral morphology.

Despite the similar moment arms, acromial deltoid moment production potential is different between the gorilla and human model (Figure 8). Therefore, these differences are related to the soft tissue properties. When scapula rotation is prohibited, humans are unable to lift their arm above 90° (Inman et al., 1944). Lucas (1973) suggested the reason for this to be the force–length relationship of the acromial deltoid. Without scapular rotation, Lucas (1973) proposed that the fibres of the human deltoid would not be able to shorten any further and therefore not produce force, beginning at approximately 90° of glenohumeral elevation. He further suggested that the scapular rotation mechanism prevents this problem. As the scapula rotates cranially, the acromion process of the scapula (deltoid origin) shifts medially, away from the humeral insertion. Thus, a certain distance between origin and insertion points is maintained as glenohumeral abduction occurs, keeping the deltoid muscle fibres closer to their optimal muscle fibre length and allowing the deltoid to maintain its moment production potential. Because differences in scapular rotation may exist between humans and gorillas (see Introduction), but the magnitude and nature of these differences remain unclear, our models compared joint function without scapular rotation. In line with expectations, the human model predicted a loss of moment production capacity at about 100° abduction (Figure 8). Our results suggest that such a scapular rotation mechanism may be of less importance in the gorilla, as the acromial deltoid muscle fibres are able to continue producing force with the arm further overhead. These findings add further support to the idea of Tuttle and Basmajian (1977) that scapular rotation is of less importance in non‐human apes due their cranial orientation of the glenoid cavity, a configuration that is only achieved after full scapular rotation in humans.

The moment arm of the clavicular deltoid also predicts a difference in action between species (Figure 5). While moment arm results suggest a pure abductor action in human clavicular deltoid, results from the gorilla model indicate a change in action from adductor to abductor with arm elevation. Similar to the spinal deltoid, this change in action stems from a difference in muscle path (relative to location of the glenohumeral joint centre) between species. In the human model, the line of action of clavicular deltoid generally runs superior to the glenohumeral joint rotation centre, due to a lateral orientation of glenoid. In *Gorilla* however, the line of action is positioned inferior to joint centre early in glenohumeral elevation. This difference in position of the muscle path relative to the joint centre follows from the cranial orientation of glenoid and clavicle, and causes the observed negative (adductor) moment arm. With increasing elevation angles, the muscle path shifts further cranially and thereby sits superior to the joint centre after 60° of elevation, which causes the change in anatomical tendency (action). In this way, differences between gorillas and humans in clavicle and glenoid orientation affect the biomechanics of clavicular deltoid, but not in a manner that improves gorilla abductor potential.

Larson and Stern (1986) observed a similar potential to change action in the clavicular deltoid. During their studies on muscle activation, they found that the clavicular deltoid was active during swing and, to a lesser extent, during support phases of vertical climbing. Therefore, the study concluded that the clavicular portion of the deltoid is both able to elevate and retract the arm, depending on arm position. These EMG observations are consistent with our prediction that clavicular deltoid has the potential to switch between adductor and abductor action in apes, depending on glenohumeral joint angle.

### 4.3 Biomechanical performance of supraspinatus and infraspinatus

Our analysis suggests that supraspinatus is able to produce much greater normalized abduction moment in *Gorilla* than *Homo* (Figure 9). This is consistent with Miller (1932), who argued for greater supraspinatus abduction moment in non‐human apes and suggested that the enhancement would arise from the obliquely oriented scapular spine. Miller suggests a twofold enhancement: (a) an obliquely oriented scapular spine leads to a widening of the supraspinous fossa, providing a larger attachment surface to a more massive muscle capable of generating greater force, and (b) “increases the abduction power of that muscle by providing it with a more advantageous mechanical location above the head of the humerus.” The comparison of dissection data reported here and by Klein Breteler et al. (1999) yields a greater supraspinatus muscle size in *Gorilla* than *Homo*, which allows for a higher maximum isometric force capacity (Table 3 and Table S1). Other researchers have also reported comparatively large masses for the *Gorilla* supraspinatus (Bello‐Hellegouarch et al., 2013; Larson, 2015; Larson & Stern, 2013). In accordance with Miller (1932), our findings supported the idea that a more massive supraspinatus muscle, associated with a larger supraspinous fossa, contributed to comparatively greater abduction moment in *Gorilla*.

Supraspinatus moment arm is also relatively larger in the gorilla than in the human model (Figure 6), demonstrating that supraspinatus abduction moment is additionally enhanced biomechanically. Our findings support Miller's assumption of a mechanical advantageous configuration. Contrary to Miller (1932), however, differences in scapular spine orientation do not appear to drive the difference in moment arm. An oblique orientation of the scapular spine in *Gorilla* is associated with a more inferior position of the supraspinatus origin, and thus a more oblique line of action. The inferior shift in origin location reduces the distance between this muscle's line of action and the joint rotation centre, thereby reducing moment arm considerably early in arm elevation and providing only slight enhancement in later arm elevation (Figure S2). Furthermore, our sensitivity analysis indicates that a more oblique line of action, as resulting from a cranially oriented scapular spine, does not improve supraspinatus moment arm over arm elevation. Our findings suggest that musculoskeletal changes associated with an oblique scapular spine lead to a reduction in supraspinatus moment arm, rather than an increase. Lee et al. (2020) made a similar observation by estimating supraspinatus moment arm during arm abduction for a large sample of morphologically variable humans. They also found an association between a more oblique spine orientation and reduced moment arm. Therefore, we suggest that a more cranially oriented scapular spine does not biomechanically enhance supraspinatus abduction moment in gorillas.

**FIGURE 12:**
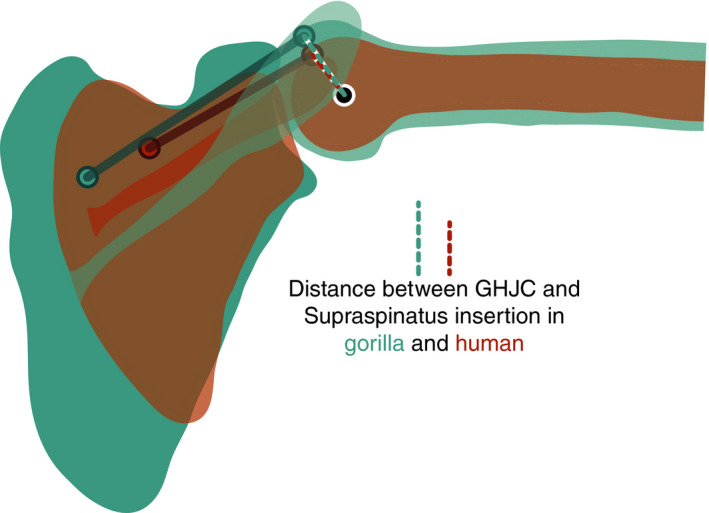
Impact of greater tubercle projection on distance between supraspinatus insertion and glenohumeral joint centre (GHJC). Shape of scapula and humerus of gorilla (in blue) and human (in brown) were superimposed to GHJC (black‐white circle). The superimposition highlights the greater distance (dashed lines) between GHJC and supraspinatus insertion site (coloured circles) in gorilla. Attachment site on the scapula has a lesser effect on the line of action. Position of the humerus relates to 95° of glenohumeral abduction

Earlier studies showed that moment arm is more sensitive to small changes in attachment sites closest to the joint rotation centre (Bates et al., 2012; Goh et al., 2017; Murray et al., 2002; O'Neill et al., 2013). In case of the supraspinatus muscle, insertion sites on the greater tubercle of the humerus are closer to joint centre than origin sites on the supraspinous fossa of the scapula. A superimposition of the shoulder bones and supraspinatus muscle attachment sites on glenohumeral joint centre (Figure 12) highlights that the distance between insertion site and joint rotation centre is greater in the gorilla than in the human model. This greater distance corresponds to a higher degree of lateral projection of greater tubercle in the gorilla. Therefore, it appears that the difference in supraspinatus moment arm between models is primarily influenced by differences in humerus morphology, specifically the radius of the humeral head and the degree of lateral projection of the greater tubercle.

The infraspinatus insertion site on the greater tubercle is increased and shifted more cranially in *Pan* and *Gorilla* compared to all other hominoids (Arias‐Martorell, 2018). The infraspinatus muscle path further differs in *Gorilla* compared to *Homo*, due to differences in scapular spine and glenoid orientation. Despite these evident differences in anatomy however, the infraspinatus moment arm is highly similar between the human and gorilla model (Figure 6). Therefore, our results indicate that skeletal modifications of scapula and humerus, and changes in muscle path do not lead to greater infraspinatus abductor leverage in *Gorilla*, compared to *Homo*.

The infraspinatus fossa is relatively smaller in gorillas than humans. While this evidently has no effect on the moment arm, it reflects a reduction in infraspinatus muscle mass and PCSA (Bello‐Hellegouarch et al., 2013; Larson, 2015). This reduction leads to a reduced maximum isometric force capacity in *Gorilla* (see *F*_max_ values in Table 3 and Table S1). As a result, abductor moment potential is less in the gorilla, than in the human model (Figure 9), despite similar moment arm values. Thus, gorilla shoulder morphology does not enhance infraspinatus abduction potential, but is instead increased in humans due to soft tissue properties.

### 4.4 Study Limitations

The musculoskeletal model presented here was designed to closely reflect the anatomical features recorded during the dissection. Therefore, this specimen‐specific model does not capture intraspecific variability present in *Gorilla*. The study individual died of old age, so some amount of muscle wasting likely occurred. However, this would not be expected to affect moment arm results markedly. Furthermore, the data informing the human model were also collected from an older individual (Klein Breteler et al., 1999), rendering the soft tissue properties more comparable. As body mass measurements were not available for the human model, moment could not be normalized by body mass or weight. Human men between 50 and 59 years were found to have masses between 63.5 and 126.7 kg with a mean of 90.5 kg (Fryar et al., 2016). The female western lowland gorilla of this study falls below this mean, with a mass of 80.5 kg. Therefore, the moment results normalized by humerus length might actually underestimate the results for the gorilla model. While great care was taken to collect the data and build the model in a way most similar as described for the human model (Klein Breteler et al., 1999; Nikooyan et al., 2011; Seth et al., 2019), different assumptions made during the model‐building process cannot be excluded.

The comparison of our measurements and model results is difficult due to a lack of studies analysing and reporting similar parameters. Until now, only Kikuchi and Kuraoka (2014) and Payne (2001) reported soft tissue properties of a gorilla shoulder. However, their data were collected from male gorillas. As size differences are great between sexes (Remis, 1998), more data from female gorillas are needed for comparison (but see Supporting Information and Table S2 for a more detailed comparison of MTU properties). Furthermore, no empirical measurements of muscle moment arms over arm ab‐/adduction (e.g. tendon travel experiments; An et al., 1984) exist for gorilla shoulder muscles. Within non‐human great apes, only Thorpe et al. (1999) reported moment arms of shoulder muscles. The authors measured deltoid moment arm statically on a male subadult chimpanzee. Their measurement of 3.3 cm falls within range of our acromial deltoid moment arm estimates. However, dynamic moment arm studies that include simultaneous measurements of arm position are needed for gorillas. We nonetheless expect validation of our model outputs from such studies, given the care taken to collecting our data and the successes with prior studies integrating experimental and theoretical calculations (Ackland et al., 2008; Brown et al., 2003; Hutchinson et al., 2015; Murray et al., 1995).

This study concentrated entirely on arm abduction potential of glenohumeral muscles. Accordingly, the analysis focused on arm elevation in the coronal plane, with long‐axis rotation kept at 0°. However, this limitation of glenohumeral joint is artificial and does not aim to reflect arm‐raising kinematics of humans or gorillas during natural movement sequences. Kinematic studies of various arboreal locomotion have shown that shoulder kinematics are highly three‐dimensional and thereby emphasized the great shoulder flexibility in hominoids (Isler, 2002, 2005; Thompson et al., 2018). Since moment arm is dependent on joint position, we can expect moment arm results to differ when glenohumeral elevation in other planes and long‐axis rotation are included into the analysis. This study further concentrates on rotation about the glenohumeral joint, which constitutes only one of the four shoulder joints that take part in human arm elevation. Additionally, the analysis was restricted to simulate moment arm and moment results of the main glenohumeral abductors. To provide greater insight into the relationship between shoulder biomechanics and morphology, future research would benefit from taking further shoulder muscles and motion around all four shoulder joints into consideration. A benefit of computer models such as ours is that such motions can be combined or separated to untangle their individual influences on biomechanical outputs.

We further want to emphasize that our results do not suggest that gorillas and humans are similarly strong. Here, we mainly tested for a mechanical advantage due to differences in shoulder configuration and additionally included soft tissue parameters to estimate muscle moment capacities. However, more variables influence muscle performance like maximum shortening velocities, fibre type composition and muscle activation (O’Neill et al., 2017; Scholz et al., 2006), which have not been considered in our analysis.

The focus of hominoid shoulder studies has long been on linking a particular pattern of shoulder morphology to advantages in glenohumeral abduction. However, our investigation did not demonstrate a clear link between these features specific for non‐human ape scapulae and stronger glenohumeral abduction capacity. While most ideas had previously focused on the deltoid, biomechanical advantages were found in the supraspinatus, but these abduction moment differences appear to be more closely related to proximal humerus morphology than to scapular morphology. Contrary to previous ideas, the very marked morphological differences between human and non‐human ape scapulae, which clearly co‐vary with function, appear to be related to arm‐lowering more than arm‐raising. Future research may benefit from focusing on how bone morphology, particularly scapula shape, affects other functions of glenohumeral rotation (especially adduction and protract/retraction outside the scapular plane) and scapular rotation—movements that are also central to arboreal locomotion and knuckle walking.

## 5 CONCLUSION

In this study, the link between shoulder morphology and biomechanical enhancement of arm‐raising in humans and gorillas was examined. We found the glenohumeral abduction potential of deltoid, supraspinatus and infraspinatus is of similar magnitude between gorillas and humans. The results cast significant doubt upon long‐standing proposals that link scapular features characteristic of arboreal primates, such as a cranially oriented scapular spine and glenoid, to biomechanical enhancement of glenohumeral abductors (Ashton & Oxnard, 1964; Corruccini & Ciochon, 1976; Miller, 1932; Roberts, 1974). Instead, our findings suggest that gorilla‐specific shoulder morphology does not enhance glenohumeral abduction moment capacity. However, our analyses demonstrate that abduction potential across arm elevation is greater in *Homo* at low amounts of arm elevation, but greater in *Gorilla* with the arm elevated above the head. These differences are mainly achieved by variation in muscle force‐production and force‐length properties. While no skeletal enhancement of deltoid abduction potential was observed in *Gorilla*, supraspinatus abduction moment capacity was found to be greater, enhanced by greater muscle force and leverage compared to *Homo*. However, improved leverage does not result from a more cranial scapular spine orientation as suggested by Miller (1932). Instead, we propose that increased lateral projection of the greater tubercle in gorilla provides a biomechanical enhancement of supraspinatus abduction moment capacity. As this study constitutes the first test of biomechanical enhancement due to shoulder morphology in hominoids, further analyses including additional shoulder muscles and joints are necessary.

## AUTHOR CONTRIBUTIONS

Julia van Beesel carried out the dissection, built the model, analysed the data and drafted the manuscript. Stephanie Melillo designed the project, organized and assisted in the dissection as well as data analysis and co‐wrote the manuscript. John Hutchinson helped with the model‐building process, with data analysis and with improving the manuscript. Jean‐Jacques Hublin provided funding and guidance for the direction of this study as well as comments on the manuscript.

## Supporting information

Fig S1

Fig S2

Fig S3

Fig S4

Fig S5

Table S1‐S2

Text S1‐S3

VideoS1

## Data Availability

The data that support the findings of this study are partially available in the article and Supporting Information and are available from the corresponding author upon reasonable request.
