# Supplementary material for: Exploring the functional morphology of the Gorilla shoulder through musculoskeletal modelling
Source: J Anat. 2021 Feb 24;239(1):207–27. doi: 10.1111/joa.13412 (PMC8197971; doi:10.1111/joa.13412)
Supplement: Supplementary file 6 — Table S1‐S2 [file JOA-239-207-s003.docx]

Supporting information tables

SI Table 1. Recalculation of maximum isometric force values used in the human model. Soft tissue property values published by Klein Breteler *et al.* (1999) were used to recalculate $F_{max}$ values. Calculations were based on Hutchinson *et al.* (2015) as described in Materials and Methods.

| Muscle unit  full name | Muscle-tendon unit (model)  abbreviation | Maximum isometric force; $F_{max}$ (N) |
| --- | --- | --- |
| Deltoideus  pars clavicularis | DeltoideusClavicle_A | 161.52 |
| Deltoideus  pars acromialis | DeltoideusScapula_M | 595.03 |
| Deltoideus  pars spinalis | DeltoideusScapula_P | 302.60 |
| Supraspinatus  anterior | Supraspinatus_A | 124.69 |
| Supraspinatus  posterior | Supraspinatus_P | 74.92 |
| Infraspinatus  superior | Infraspinatus_S | 223.27 |
| Infraspinatus  inferior | Infraspinatus_I | 238.25 |

SI Table 2. Comparative MTU properties of deltoid, supraspinatus and infraspinatus reported in this study and in the literature. If data of multiple specimens was reported, only the specimen with the most complete data or best compatibility (based on body weight, age and sex) is represented here. The asterisk labels MTU property values that are smaller relative to body mass compared to MTU properties reported in our study.

| Study | Species | Body mass (kg) | Sex | Age | Muscle | Muscle mass (kg) | Fascicle length (m) | PCSA (m²) |
| --- | --- | --- | --- | --- | --- | --- | --- | --- |
| This study | *Gorilla gorilla* | 80 | Female | 49 | Deltoideus | 0.2862 | 0.1335 (average) | 0.0020 (average) |
|  |  |  |  |  | Supraspinatus | 0.0840 | 0.0662 | 0.0012 |
|  |  |  |  |  | Infraspinatus | 0.1048 | 0.0839 | 0.0012 |
| Kikuchi and Kuraoka (2014) | *Gorilla gorilla* | - | Male | - | Deltoideus | 0.680 | 0.0969 | 0.0066 |
|  |  |  |  |  | Supraspinatus | 0.172 | 0.0804 | 0.0020 |
|  |  |  |  |  | Infraspinatus | 0.2798 | 0.1098 | 0.0024 |
| Payne (2001) | *Gorilla gorilla* | 130 | Male | 35 | Deltoideus | 0.6008 | 0.0631* | 0.008979 |
|  |  |  |  |  | Infraspinatus | 0.2677 | 0.1137* | 0.00222 |
| Carlson (2006) | *Pan troglodytes* | 54.7 | Female | 48 | Deltoideus | 0.1727* (wet) | 0.094 | 0.00174 |
|  |  |  |  |  | Supraspinatus | 0.0382* | 0.02* | 0.00177 |
|  |  |  |  |  | Infraspinatus | 0.0742 | 0.042* | 0.00167 (wet) |
| Kikuchi *et al.* (2012) | *Pan troglodytes* | 32.2 | Female | Adult | Deltoideus | 0.18088 | 0.0543 | 0.0030 |
|  |  |  |  |  | Supraspinatus | 0.0338 | 0.0406 | 0.0007 |
|  |  |  |  |  | Infraspinatus | 0.06243 | 0.0598 | 0.0009 |
| Oishi *et al.* (2009) | *Pan troglodytes* | 30.2 | Female | 11 | Deltoideus | 0.2054 | 0.0867 | 0.0022 |
|  |  |  |  |  | Supraspinatus | 0.0526 | 0.038 | 0.0013 |
|  |  |  |  |  | Infraspinatus | 0.1103 | 0.064 | 0.0016 |
| Thorpe *et al.* (1999) | *Pan troglodytes* | 37 | Male | 6 | Deltoideus | 0.276 | 0.083 | 0.0031 |
|  |  |  |  |  | Infraspinatus | 0.116 | 0.068 | 0.0016 |
