# Supplementary material for: Exploring the functional morphology of the Gorilla shoulder through musculoskeletal modelling
Source: J Anat. 2021 Feb 24;239(1):207–27. doi: 10.1111/joa.13412 (PMC8197971; doi:10.1111/joa.13412)
Supplement: Supplementary file 7 — Text S1‐S3 [file JOA-239-207-s002.docx]

Electronic Supporting Information

SI Text S1. Sensitivity analysis

Positions of muscle attachment influence muscle paths, and therefore muscle moment arms. In order to compare muscle moment arm results of the gorilla model from this study and the human model of Seth *et al.* (2019), we modelled attachments similar to the human model, while still reflecting MTU geometry captured during the gorilla dissection. However, supra- and infraspinatus are each separated into two subunits in the human model, but only modelled as one in the gorilla model (Table 1). While insertion points of both subunits are similar for each muscle, the distance between origin positions is marked. We therefore altered the origin points of both supra- and infraspinatus in the here presented gorilla musculoskeletal model to replicate the positions in the human model. The results of the sensitivity analysis are presented in SI Figure 2 for supraspinatus and in SI Figure 3 for infraspinatus.

SI Text S2. MTU geometry reconstruction

Muscle attachments: While taking muscles off during the dissection, the circumference of each muscle-tendon unit (MTU) was labelled using coloured pins (SI Figure 4) on all bones they were attached to. Additionally, different coloured pins were used to label anatomical landmarks previously identified. Photographs were taken for later reference. Surface scans, using a structured-light surface scanner (Artec Space Spider with Artec Studio 12 software, Artec 3D, Luxembourg), were taken for attachment areas of each MTU, with both origin and insertion sites in one scan. Multiple scans were taken (e.g. overview and detail scans) and fused into one surface using the scanning software (Artec Studio 12 software, Artec 3D, Luxembourg). In Avizo software (version 9.3.0, Visualization Sciences Group, Burlington, MA, USA), the labelled anatomical landmarks were used for a landmark-based affine registration of the scan surface to the bone surfaces of the CT scan. Here, each surface scan was registered to the space of each bone containing attachment areas (in the example of SI Figure 4, the acromial and spinal deltoid surface scan was duplicated and one registered to the scapula surface, one registered to the humerus surface). In Geomagic Studio® (version 2013, RSI 3D-Systems), the surface scans were made transparent and the pins on the surface scans surrounding the muscle attachment areas were used to label these areas on the bone surfaces. The attachment areas were extracted from the bone surfaces and the centroids were calculated in Rhinoceros software (version 6, McNeel Europe, Spain).

Muscle paths: Before taking the muscles off during the dissection, the midline of each MTU was labelled as well as anatomical landmarks using coloured pins (SI Figure 5 A). Photographs were taken for later reference. Surface scans were taken for three different arm positions (abducted, intermediate and adducted). For each arm position, a surface was created in the scanning software. The surface was then registered to the bone surface the captured MTU was originating from (SI Figure 5 B; all muscles were registered to the scapula surface, except the clavicular deltoid, which was registered to the clavicle surface) in Avizo software. The registered path surfaces were imported into OpenSim and previously calculated muscle attachments were inserted into the model. Specific path points and wrapping surfaces used in the human model of Seth *et al.* (2019) were inserted. The resulting MTU paths were evaluated using the labelled midline visible in the path surfaces (SI Figure 5 C) for the three different arm positions. Path points and wrapping surfaces were adjusted to restrict path points to the labelled midlines.

SI Text S3. Comparison of MTU properties

The comparison of MTU properties measured in this study is difficult due to a lack of data reported for female gorillas. Females are not miniature males, as the segment masses relative to body mass differ between sexes (Zihlman, 1992). Zihlman and McFarland (2000) found that male gorillas have relatively heavier forelimbs compared to females. Moreover, the deltoid muscle was found to be lighter in females relative to total muscle mass compared to male gorillas. Therefore, we expect muscle masses relative to body mass to be smaller for female than male gorillas. Muscle fascicle length would be expected to relate to segment length (which might not scale with body mass). However, this data is usually not reported. Therefore, muscle fascicle length and PCSA (based on both muscle mass and fascicle length) are compared relative to body mass. Unfortunately, Kikuchi and Kuraoka (2014) did not report the body mass of their gorilla specimen, but MTU property data is similar to data reported by Payne (2001), with the exception of deltoid fascicle length and PCSA (SI Table 2).

Muscle masses relative to body mass are smaller in the female than the male gorilla (Payne, 2001) as expected, as well as relative PCSA values (SI Table 2). However, relative fascicle lengths are greater in the female gorilla, with a pronounced difference between deltoid fascicle lengths. This could be a result of relatively bigger segments (shoulder and upper arm), or of an overestimation of fascicle length of the female gorilla. Additionally, both studies that report MTU properties of gorillas do not give details where fascicle length data was collected across the deltoid muscle. As fascicle lengths differ highly between the acromial and the other two deltoid MTUs (Table 3), the knowledge about sample position is crucial for comparison. If only relative fascicle length of the acromial deltoid MTU is compared to data reported by Payne (2001), differences are less pronounced. However, as longer fascicle lengths lead to smaller PCSA and maximum isometric force values, an overestimation of force and moment capacity for the gorilla model is unlikely.

We additionally included MTU property data of *Pan troglodytes* into SI Table 2 for comparison. However, it is important to bear in mind that geometrical relationships do not scale with size in African apes. Species with increased body size become relatively shorter and more stout, which might result in smaller MTU properties relative to body mass (see Isler (2005) and references therein). Indeed, relative MTU properties are smaller in the female gorilla compared to the data reported by the other studies (with four exceptions, labelled by an asterisk in SI Table 2). Deltoid and supraspinatus muscles masses reported by Carlson (2006) are relatively smaller compared to the female gorilla (relative masses of infraspinatus are nearly identical). The differences could be related to the high age of the *Pan* specimen, and therefore age related loss of muscle masses. Similarly, relative muscle fascicle lengths of supra- and infraspinatus are shorter in that specimen, but not of deltoid.

SI Figure Capitations

SI Figure 1. Absolute moment arm changes of deltoid muscle over glenohumeral elevation. The grey solid line separates MTUs acting as abductors (positive moment arms) from those acting as adductors (negative moment arms).

SI Figure 2. Sensitivity analysis of supraspinatus absolute moment arms. Supraspinatus muscle is represented by two subunits in the human model, with differing scapular attachments. Similar muscle origin positions of both subunits were remodelled in the gorilla model to analyse the influence on moment arm. For muscle abbreviations see SI Table 1. The grey solid line separates muscle units acting as abductors (positive moment arms) from those acting as adductors (negative moment arms).

SI Figure 3. Sensitivity analysis of infraspinatus absolute moment arms. Infraspinatus muscle is represented by two subunits in the human model, with differing scapular attachments. Similar muscle origin positions of both subunits were remodelled in the gorilla model to analyse the influence on moment arm. For muscle abbreviations see SI Table 1. The grey solid line separates muscle units acting as abductors (positive moment arms) from those acting as adductors (negative moment arms).

SI Figure 4. Reconstruction of acromial and spinal deltoid attachment surfaces on the humerus (A) and scapula (B). Left: Photographs taken to highlight the position of different coloured pins on the bone and to help reconstructing the surface scans. White pins label attachment area of both MTUs on the humerus and of acromial deltoid on the scapula, blue pins label spinal deltoid attachment area on the scapula. Yellow and green pins label anatomical landmarks. Middle: Registration of surface scan (red) to bone surface from CT (dark grey) based on anatomical landmarks. Right: Attachment areas of spinal deltoid (green) and acromial deltoid (blue) on the humerus and scapula.

SI Figure 5. Muscle path reconstruction of the acromial and spinal deltoid. A. Surface scans were taken for three different arm positions (adducted, intermediate and abducted). Coloured pins label the midline of acromial (white pints) and spinal (blue pins) deltoid, whereas yellow pins label anatomical landmarks. B. Anatomical landmarks were applied to the surface scan (left) and bone surface of CT scan (middle) and used to register the surface to the CT scan (right). C. Registered surface scans (orange) were inserted into the musculoskeletal model and the glenohumeral joint was manipulated to mirror the arm positions of the three different surface scans (abducted, left; intermediate, middle; adducted, right). Pins labelling the midline of each MTU (red) were visible and aided in evaluating muscle paths and assessing the wrapping surfaces (blue).

References

Carlson, K. J. (2006) Muscle architecture of the common chimpanzee (*Pan troglodytes*): perspectives for investigating chimpanzee behavior. *Primates,* **47**(3), 218-229. doi:10.1007/s10329-005-0166-4

Hutchinson, J. R., Rankin, J. W., Rubenson, J., Rosenbluth, K. H., Siston, R. A., and Delp, S. L. (2015) Musculoskeletal modelling of an ostrich (*Struthio camelus*) pelvic limb: influence of limb orientation on muscular capacity during locomotion. *PeerJ,* **3**, e1001. doi:10.7717/peerj.1001

Isler, K. (2005) 3D-kinematics of vertical climbing in hominoids. *American Journal of Physical Anthropology,* **126**(1), 66-81. doi:10.1002/ajpa.10419

Kikuchi, Y., and Kuraoka, A. (2014) Differences in Muscle Dimensional Parameters Between Non-Formalin-Fixed (Freeze-Thawed) and Formalin-Fixed Specimen in Gorilla (*Gorilla gorilla*). *Mammal Study,* **39**(1), 65-72, 68. doi:10.3106/041.039.0101

Kikuchi, Y., Takemoto, H., and Kuraoka, A. (2012) Relationship between humeral geometry and shoulder muscle power among suspensory, knuckle-walking, and digitigrade/palmigrade quadrupedal primates. *Journal of Anatomy,* **220**(1), 29-41. doi:10.1111/j.1469-7580.2011.01451.x

Klein Breteler, M. D., Spoor, C. W., and van der Helm, F. C. T. (1999) Measuring muscle and joint geometry parameters of a shoulder for modeling purposes. *Journal of biomechanics,* **32**(11), 1191-1197. doi:10.1016/S0021-9290(99)00122-0

Oishi, M., Ogihara, N., Endo, H., Ichihara, N., and Asari, M. (2009) Dimensions of forelimb muscles in orangutans and chimpanzees. *Journal of Anatomy,* **215**(4), 373-382. doi:10.1111/j.1469-7580.2009.01125.x

Payne, R. C. (2001). *Musculoskeletal adaptations for climbing in hominoids and their role as exaptions for the acquisition of bipedalism.* University of Liverpool, Retrieved from http://ethos.bl.uk/ProcessSearch.do?query=367705 EThOS database.

Seth, A., Dong, M., Matias, R., and Delp, S. (2019) Muscle Contributions to Upper-Extremity Movement and Work From a Musculoskeletal Model of the Human Shoulder. *Frontiers in Neurorobotics,* **13**(90). doi:10.3389/fnbot.2019.00090

Thorpe, S. K. S., Crompton, R. H., Günther, M. M., Ker, R. F., and McNeill Alexander, R. (1999) Dimensions and moment arms of the hind- and forelimb muscles of common chimpanzees (*Pan troglodytes*). *American Journal of Physical Anthropology,* **110**(2), 179-199. doi:10.1002/(SICI)1096-8644(199910)110:2<179::AID-AJPA5>3.0.CO;2-Z

Zihlman, A. L. (1992) Locomotion as a life history character: the contribution of anatomy. *Journal of human evolution,* **22**(4), 315-325. doi:10.1016/0047-2484(92)90062-E

Zihlman, A. L., and McFarland, R. K. (2000) Body mass in lowland gorillas: A quantitative analysis. *American Journal of Physical Anthropology,* **113**(1), 61-78. doi:10.1002/1096-8644(200009)113:1<61::AID-AJPA6>3.0.CO;2-H
